# Ectopic expression of Klotho in fibroblast growth factor 23 (FGF23)-producing tumors that cause tumor-induced rickets/osteomalacia (TIO)

**DOI:** 10.1016/j.bonr.2018.100192

**Published:** 2018-12-31

**Authors:** Yuka Kinoshita, Yuichi Takashi, Nobuaki Ito, Shiro Ikegawa, Hiroyuki Mano, Tetsuo Ushiku, Masashi Fukayama, Masaomi Nangaku, Seiji Fukumoto

**Affiliations:** a Division of Nephrology and Endocrinology, Department of Medicine, The University of Tokyo Hospital, Tokyo 113-8655, Japan; b Diabetes Therapeutics and Research Center, Institute of Advanced Medical Sciences, Tokushima University, Tokushima 770-8503, Japan; c Laboratory for Bone and Joint Diseases, RIKEN Center for Integrative Medical Sciences, Tokyo 108-8639, Japan; d National Cancer Center Research Institute, Tokyo 104-0045, Japan; e Department of Pathology and Diagnostic Pathology, Graduate School of Medicine, The University of Tokyo, Tokyo 113-8655, Japan; f Fujii Memorial Institute of Medical Sciences, Tokushima University, Tokushima 770-8503, Japan

**Keywords:** Tumor-induced osteomalacia (TIO), Klotho, Fibroblast growth factor 23 (FGF23), FGF receptor (FGFR), Hypophosphatemia

## Abstract

Tumor-induced rickets/osteomalacia (TIO) is a rare paraneoplastic syndrome caused by tumors that ectopically express fibroblast growth factor 23 (FGF23). FGF23 is a bone-derived hormone that regulates serum phosphate concentrations. Patients with TIO develop hypophosphatemic rickets/osteomalacia due to FGF23 excess and suffer from symptoms such as leg deformities, bone pain, skeletal muscle myopathy, and multiple fractures/pseudofractures. Usually, successful surgical removal of the causative tumors normalizes serum FGF23 and phosphate concentrations in patients with TIO. Most FGF23-producing tumors associated with TIO are histologically called phosphaturic mesenchymal tumor, mixed connective tissue variant (PMTMCT). The precise mechanism by which these tumors ectopically overproduce FGF23 outside of bone is yet to be clarified. Therefore, we performed an RNA sequencing analysis of a PMTMCT that was found in the left parotid gland of a patient with TIO. Among the upregulated genes, we focused on *Klotho*, the protein product of which is a single pass transmembrane protein that works along with an FGF receptor 1c as a receptor complex for FGF23. Subsequent histological analysis confirmed the ectopic expression of Klotho in other PMTMCTs. From these results, we assume that the ectopic expression of Klotho in PTMMCTs enables a positive feedback loop in FGF23 production *via* the activation of FGF receptor 1c and exacerbates disease manifestations in TIO.

## Highlights

• Klotho is ectopically expressed in the FGF23-producing mesenchymal tumors.
• Klotho enables the activation of the FGFR signaling pathway in PMTMCTs.
• Klotho enables the autocrine/paracrine effects of FGF23 in PMTMCTs.

## 1 Introduction

Tumor-induced rickets/osteomalacia (TIO) is a rare paraneoplastic syndrome caused by tumors that overproduce fibroblast growth factor 23 (FGF23) (Shimada et al., 2001; Kumar, 2000). FGF23 is a bone-derived hormone that regulates serum phosphate concentrations (Fukumoto and Martin, 2009). FGF23 reduces phosphate reabsorption in the proximal renal tubules by downregulating sodium-phosphate cotransporters. FGF23 also decreases phosphate absorption in the intestine by reducing serum active vitamin D: 1,25‑dihydroxyvitamin D [1,25(OH)_2_D] levels (Shimada et al., 2004). Therefore, chronic FGF23 excess leads to vitamin D-resistant hypophosphatemia. Adult patients with TIO suffer from symptoms such as bone pain, skeletal muscle myopathy, and multiple fractures/pseudofractures (Minisola et al., 2017). Additionally, growth retardation and leg deformities are common features of patients with childhood-onset TIO (Imel et al., 2006).

Historically, McCance reported a patient with osteomalacia with resistance to vitamin D in 1947, which is considered to be the first reported case of TIO (McCance, 1947). Weidner and Santa Cruz described 17 cases of mesenchymal tumors that caused osteomalacia or rickets in 1987 (Weidner and Santa Cruz, 1987). Later, it was revealed that FGF23 excess is responsible for the pathogenesis of TIO (Shimada et al., 2001; Kumar, 2000). Moreover, FGF23 excess also leads to several congenital hypophosphatemic diseases, such as autosomal dominant hypophosphatemic rickets (ADHR: OMIM #193100) (ADHR Consortium, 2000) and X-linked hypophosphatemia (XLH; OMIM #307800) (The HYP Consortium, 1995).

Most causative tumors for TIO are benign and located in bone or soft tissue (Folpe et al., 2004), except for a few cases of TIO caused by malignancies such as colorectal, ovarian, prostate, and thyroid cancers (Lin et al., 2014; Leaf et al., 2013; Mak et al., 2012; Abate et al., 2016). These mesenchymal tumors are histologically termed phosphaturic mesenchymal tumor, mixed connective tissue variant (PMTMCT). The typical features of PMTMCT are the dense proliferation of spindle-shaped cells, myxoid areas, chondroid or osteoid-matrix containing flocculent calcification, osteoclast-like giant cells, and blood vessels (Folpe et al., 2004). The majority of PMTMCTs are histologically benign, but a relatively small number of malignant PMTMCTs with distant metastases have been reported (Morimoto et al., 2014; Nair et al., 2017; Yavropoulou et al., 2018).

FGF23 is physiologically produced in osteocytes/osteoblasts and binds to a complex of FGF receptor 1c (FGFR1c) and αKlotho (referred to as Klotho in this manuscript) to serve its function (Urakawa et al., 2006; Kurosu et al., 2006). Klotho is a single-pass transmembrane protein that shares sequence homology with family I beta-glycosidases (Matsumura et al., 1998). In human, the expression of Klotho is restricted to specific organs such as the kidney, prostate, placenta, and parathyroid glands. Considering the ubiquitous expression of FGFR1c, it is likely that Klotho enables the site-specific action of FGF23 (Urakawa et al., 2006). *Klotho*-deficient mice show FGF23 resistance with a 2000-fold increase in serum FGF23 concentrations compared to those in wild-type mice (Kuro-o et al., 1997). Moreover, because of the loss of FGF23 function, *Klotho*-deficient mice show similar phenotypes to those of *Fgf23*-null mice, such as ectopic calcification, elevated serum 1,25(OH)_2_D concentrations, and hyperphosphatemia (Kurosu et al., 2006). Therefore, it is widely accepted that Klotho plays an essential role in FGF23 signaling.

The regulatory mechanism of FGF23 production in osteocytes/osteoblasts is not fully understood. Animal and human studies have shown that specific conditions, such as treatment with active vitamin D (Kolek et al., 2005; Yamamoto et al., 2010; Hansen et al., 2012; Sprague et al., 2015), phosphate overload (Hori et al., 2016; Perwad et al., 2005; Antoniucci et al., 2006; Ferrari et al., 2005), and uremia (Stubbs et al., 2007), upregulate *FGF23* transcription in bone and elevate circulating FGF23 concentrations. Additionally, previous studies of inherited FGF23-related hypophosphatemic diseases have shown the involvement of several gene products in *FGF23* transcription. For example, inactivating mutations in the *phosphate-regulating endopeptidase homolog*, *X-linked* (*PHEX*) gene lead to the upregulation of *FGF23* production in patients with XLH, which is the most prevalent form of genetic FGF23-related hypophosphatemic rickets (Liu et al., 2003).

So far, the precise mechanism of ectopic FGF23 production in tumors that cause TIO is unknown. Recent studies have shown the presence of *FN1*-*FGFR1* or *FN1-FGF1* fusion genes in several tumors that are causative for TIO. However, the function of these fusion gene products remains unknown (Lee et al., 2015; Lee et al., 2016). Therefore, to examine the regulatory mechanism of FGF23 production in TIO-related tumors, we performed an RNA sequencing analysis using the responsible tumor in the parotid gland from a patient with TIO. Subsequent investigation confirmed the ectopic expression of Klotho in FGF23-producing tumors, which suggests the involvement of Klotho in the pathogenesis of TIO.

## 2 Subjects and methods

### 2.1 Subjects

We performed RNA sequencing analysis using RNA extracted from a tumor and the adjacent normal tissue in the parotid gland of a patient with TIO (Takashi et al., 2017) (case #1). The brief medical history of the patient is as follows: the patient was a 77-year-old man who had been suffering from progressive pain in the back and the hip joints for eight years. Laboratory data showing hypophosphatemia and elevated serum FGF23 concentrations led to the diagnosis of FGF23-related hypophosphatemic osteomalacia. An ^18^F-FDG-PET/CT scan revealed a tumor in the left parotid gland. Higher FGF23 concentration in the left external jugular vein compared to other sites indicated that the tumor overproduced FGF23. When the tumor was surgically removed, the patient's serum phosphate concentration returned to the normal range. The tumor was histologically diagnosed as a PMTMCT with positive FGF23 staining.

We subsequently enrolled 12 patients with TIO whose formalin-fixed, paraffin-embedded (FFPE) tumor samples were available (cases #2–#13). Table 1 summarized the characteristics of patients and the location of the tumors. All the patients were treated with a combination of alfacalcidol and oral phosphate salts. Biochemical parameters including serum albumin, calcium, phosphate, alkaline phosphatase (ALP), creatinine (Cr), intact parathyroid hormone (iPTH), 1,25(OH)_2_D, and urine phosphate and creatinine were collected from the medical record. These parameters were measured before the surgery. Serum FGF23 levels were evaluated using an FGF23 ELISA KIT (Kainos, Tokyo, Japan) that detects only full-length FGF23 (Endo et al., 2008). Although the reference range for serum FGF23 is 10–50 pg/mL for healthy subjects without chronic kidney disease, FGF23 levels more than 30 pg/mL are considered abnormally high in patients with hypophosphatemia and leads to the diagnosis of FGF23-related hypophosphatemia (Endo et al., 2008). Serum calcium concentrations were corrected by albumin if albumin was <4.0 mg/dL. The reference ranges for adults are 8.8–10.1 mg/dL for serum corrected calcium, 2.7–4.6 mg/dL for serum phosphate, 106–332 U/L for serum ALP, 10–65 pg/mL for serum iPTH, 20–60 pg/mL for serum 1,25(OH)_2_D, and 0.65–1.07 mg/dL (men) and 0.46–0.79 mg/dL (women) for serum creatinine. The tubular maximum reabsorption of phosphate per unit of glomerular filtrate (TmP/GFR) was calculated using serum phosphate, serum creatinine, urine phosphate, and urine creatinine concentrations. The reference range for TmP/GFR is 2.3–4.3 mg/dL.

**Table 1:** Patient characteristics.

Case	Age^a^/sex	Location of the tumor	Serum parameters^b^							TmP/GFR^b^ (2.3–4.3 mg/dL)
			Calcium (8.8–10.1 mg/dL)	Phosphate (2.7–4.6 mg/dL)	Creatinine (M, 0.65–1.07 mg/dL; F, 0.46–0.79 mg/dL)	ALP (106–332 U/L)	iPTH (10–65 pg/mL)	FGF23 (10–50 pg/mL)	1,25(OH)_2_D (20–60 pg/mL)	
1	77M	Left parotid gland	8.8	3.3	0.72	515	80	187	70.7	1.7
2	53F	Right external ear canal	8.4	2.7	0.36	1880	47	464	36.4	2.0
3	51M	Left sole	8.6	2.0	1.07	1765	22	893	52.7	0.9
4	36F	Left nasal cavity	8.9	2.3	0.52	270	45	142	19.1	1.6
5	64F	Right iliac bone	8.5	2.2	0.46	832	77	117	26.7	1.7
6	69M	Right deltoid muscle	9.0	2.2	0.84	292	34	488	22.8	1.8
7	53M	Right femur	9.3	2.8	1.16	478	220	1475	5.1	1.2
8	41F	Left maxillary sinus	8.6	2.3	0.66	610	83	423	65.5	1.2
9	59M	Left femur	9.0	2.0	0.57	1242	88	100	116.0	1.1
10	69M	Right femur	8.2	2.3	0.61	834	37	3280	35.9	1.4
11	45M	Right femoral neck	8.7	1.5	0.73	679	82	166	18.3	1.2
12	63M	First lumbar vertebral body	9.0	2.5	0.94	404	71	5211	47.3	1.5
13	59M	Palate	8.2	1.4	0.65	712	140	94	53.0	0.8
		Average ± SD (all)	8.8 ± 0.4	2.3 ± 0.5	0.71 ± 0.23	809 ± 519	79 ± 52	1003 ± 1543	43.8 ± 29.1	1.4 ± 0.4
		Average ± SD (Men, n = 9)	8.8 ± 0.4	2.2 ± 0.6	0.81 ± 0.21^c^	769 ± 467	86 ± 62	1322 ± 1785	46.9 ± 32.9	1.3 ± 0.3
		Average ± SD (Women, n = 4)	8.6 ± 0.2	2.4 ± 0.2	0.5 ± 0.13^c^	898 ± 694	63 ± 20	267 ± 182	36.9 ± 20.3	1.6 ± 0.3

a Age at the operation.

b Data prior to the operation, reference range for each parameter is given in brackets.

c Significant difference between male and female patients.

This study was approved by the institutional review board of the University of Tokyo. We obtained written informed consent for the RNA sequencing and opt-out consent for both the immunohistochemical and RT-PCR analyses of tumor samples.

### 2.2 RNA sequencing of the parotid tumor

Total RNA was extracted from the fresh frozen parotid gland tumor and the adjacent normal parotid gland tissue using the NucleoSpin® RNA kit (Macherey-Nagel, Duren, Germany).

Paired-end sequencing libraries were constructed using the TruSeq RNA library Prep Kit v2 (Illumina, San Diego, CA). We sequenced the libraries on a MiSeq system (Illumina, San Diego, CA) using the 300-cycles MiSeq Reagent Kits v2 (Illumina, San Diego, CA). Data were analyzed on a CLC genomic workbench v8 (CLC bio Japan, Tokyo, Japan).

For RT-PCR, total RNA was reverse transcribed to cDNA using the PrimeScript® RT Master Mix (Takara Bio, Shiga, Japan). We amplified 100–300 base products of *FGF23*, *Klotho*, *FGFR1c*, and *GAPDH* cDNA using the SapphireAmp® Fast PCR Master Mix (Takara Bio, Shiga, Japan). The primer sets were as follows: 5′-TCTTTGCTTTGGACCCACCT-3′ and 5′-CCACTCGAAACCATCCATGA-3′ for *Klotho*, 5′-ACTGCTGGAGTTAATACCAC-3′ and 5′-TTCCAGAACGGTCAACCAT-3′ for *FGFR1c*, and 5′-TGGCACCGTCAAGGCTGAGA-3′ and 5′-CCAGCATCGCCCCACTTGAT-3′ for *GAPDH*. We purchased the primer sets for FGF23 from TaKaRa Bio (HA181049). The primer sets designed to detect fusion genes were as follows: 5′-TCGGGCCCAGATAACAGGATAC-3′ and 5′-GCCTCCAATTCTGTGGTCAGGT-3′ for the *FN1*-*FGFR1* fusion gene and 5′-TCGGGCCCAGATAACAGGATAC-3′ and 5′-TGCTTTCTGGCCATAGTGAGTC-3′ for the *FN1*-*FGF1* fusion gene. PCR conditions were as follows: 1 min at 94 °C, followed by 35 cycles of 5 s at 98 °C, 5 s at 58 °C, and 5 s at 72 °C, with a final extension for 3 min at 72 °C. PCR products were electrophoresed on a 1% agarose gel containing ethidium bromide and visualized with UV light.

### 2.3 Immunohistochemistry of the tumor samples

Immunohistochemical (IHC) staining was performed using the Ventana BenchMark automated immunostainer (Ventana benchmark; Ventana Medical Systems Inc., Tucson, AZ) according to the manufacturer's instructions. The primary antibodies used in this study were an anti-human Klotho monoclonal antibody that detects 55–261 amino acids of human Klotho (KM2076, Trans Genic Inc., Kobe, Japan) and an anti-phospho-Erk1/2 antibody that recognizes phospho-p44/42 MAPK (#4370, Cell Signaling Technology Japan, Tokyo, Japan).

### 2.4 Analysis of serum Klotho concentrations in the patients

A human soluble alpha-Klotho assay kit (IBL, Gunma, Japan) was used to measure the serum Klotho concentrations in 11 out of 13 patients.

### 2.5 Statistical analysis

The relationships between each biochemical parameter of the patients and the relationships between serum FGF23 and serum Klotho concentrations were analyzed using JMP® Pro14.0 (SAS Institute Japan Ltd., Tokyo, Japan). *p* values <0.05 were considered statistically significant.

## 3 Results

### 3.1 Patient characteristics

We analyzed nine male patients and four female patients with TIO in our study. Even though all the patients were treated with a combination of alfacalcidol and oral phosphate salts, seven and ten of 13 patients showed hypocalcemia and hypophosphatemia, respectively (Table 1). Serum ALP levels were elevated in 10 patients. Eight patients showed secondary hyperparathyroidism. TmP/GFR levels were lower than the reference range in all the patients, suggesting that there was an increase in renal phosphate wasting as a result of FGF23 excess. In fact, there was a significant positive correlation between serum phosphate levels and TmP/GFR levels (r = 0.6179, *p* = 0.0244). Additionally, there was a significant negative correlation between serum corrected calcium and ALP (r = −0.4018, *p* = 0.0322). We did not find any significant correlation between serum FGF23 and other parameters. At the same time, there was no significant difference in parameters between male and female patients other than serum creatinine levels (Table 1).

### 3.2 RNA sequencing analysis of the tumor in the parotid gland

**Fig. 1:**
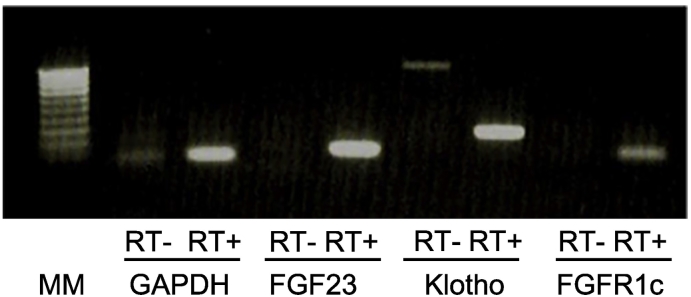
RT-PCR analysis of a tumor in a parotid gland. Gel-electrophoresis of RT-PCR products showed the presence of *FGF23*, *Klotho*, *FGFR1c* mRNA in the tumor. MM, molecular weight marker; RT, reverse transcription.

We detected several upregulated and downregulated genes in the parotid gland tumor compared to those in the adjacent normal tissue. Table 2 shows a list of representative upregulated genes in the tumor. We could not identify any significant fusion genes that involve *FGFR1* or *FGF1*. The RT-PCR analysis confirmed the presence of *FGF23*, *Klotho*, and *FGFR1c* mRNA in the tumor (Fig. 1).

**Table 2:** Representative upregulated genes in an FGF23-producing tumor in the parotid gland.

Name	RPKM^a^		Fold change
	Control	Tumor	
*DMP1*	0.9545	2411.07	2345
*FGF1*	0.0298	67.6582	2107
*FGF23*	0.1497	207.605	1288
*MEPE*	5.2285	7159.17	1271
*SOST*	2.4226	3257.41	1248
*SFRP4*	1.5490	1976.49	1185
*Klotho*	0.4545	445.978	911
*SPP1*	12.9661	11,899.7	852
*ACP5*	0.3211	236.662	684
*PHEX*	0.1478	104.634	657
*MMP9*	2.9010	1659.15	531

DMP1, dentin matrix protein 1; FGF1, fibroblast growth factor 1; FGF23, fibroblast growth factor 23; MEPE, matrix extracellular phosphoglycoprotein; SOST, sclerostin; SFRP4, secreted frizzled-related protein 4; SPP1, secreted phosphoprotein 1; ACP5, acid phosphatase 5, tartrate resistant; PHEX, phosphate-regulating endopeptidase homolog, X-linked; MMP9, matrix metalloproteinase 9.

a RPKM, reads per kilobase of exon per million mapped reads.

### 3.3 Immunohistochemistry of the causative tumors in TIO

**Fig. 2:**
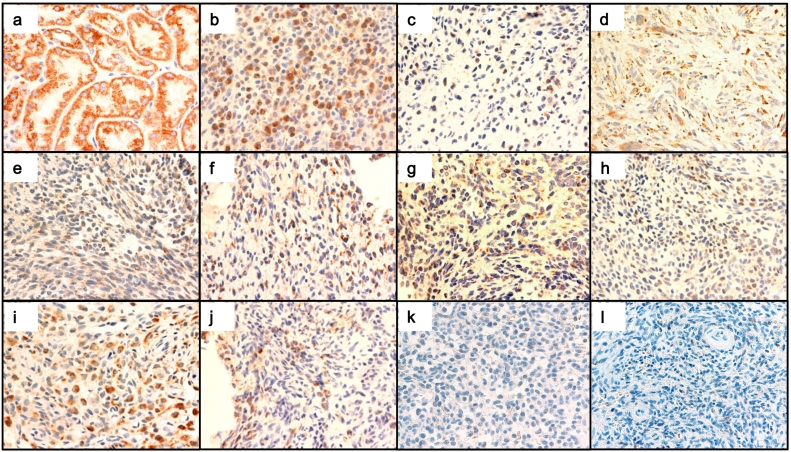
Klotho staining of FGF23-producing tumors. The spindle-shaped tumor cells, which are considered the principal source of FGF23 in PMTMCTs, were positive for Klotho staining in nine of 13 patients. Shown are the images of tumors with positive Klotho staining (b, case #1; c, case #2; d, case #3; e, case #4; f, case #5; g, case #7; h, case #9; i, case #10; j, case #13) and those with negative Klotho staining (k, case #8; l, case #12), along with a positive control of renal tubular cells (a). Original magnification for all photomicrographs is ×400.

**Fig. 3:**
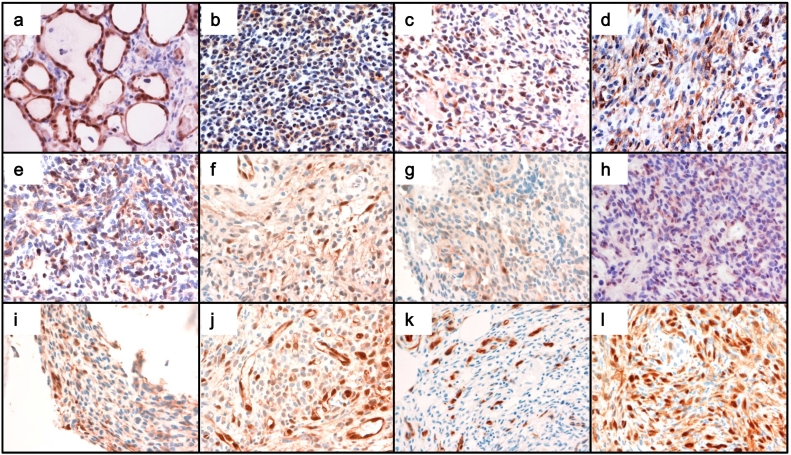
Phospho-Erk staining of FGF23-producing tumors. Tumor cells were positive for phospho-Erk1/2 staining in 12 of 13 patients (b, case #1; c, case #2; d, case #3; e, case #4; f, case #5; g, case #6; h, case #7; i, case #8; j, case #9; k, case #12; l, case #13) as well as the positive control of the thyroid follicular cells (a). Original magnification for all photomicrographs is ×400.

Among 13 tumors from patients with TIO, nine tumors were positive for IHC staining for Klotho (Table 3). In these tumors, spindle-shaped tumor cells, which are considered the principal source of FGF23 in TIO, were positive for Klotho staining. Fig. 2 shows the images of tumor cells positive for Klotho staining (Fig. 2b–j) and those negative for Klotho staining (Fig. 2k and l), along with a positive control of renal tubular cells (a). Additionally, 11 of 13 tumors were positive for phospho-Erk1/2 staining (Fig. 3b–l) as shown in the thyroid follicular cells (Fig. 3a).

**Table 3:** Summary of immunohistochemical and RT-PCR analyses of tumors.

Case	Klotho		p-Erk
	IHC	RT-PCR	IHC
1	+	+	+
2	+	N.E.	+
3	+	+	+
4	+	N.E.	+
5	+	+	+
6	−	N.E.	+
7	+	N.E.	+
8	−	N.E.	+
9	+	N.E.	+
10	+	N.E.	N.E.
11	−	−	N.E.
12	−	N.E.	+
13	+	N.E.	+

IHC, Immunohistochemistry; +, positive; −, negative; N.E., not examined.

### 3.4 Analysis of serum Klotho concentrations

**Fig. 4:**
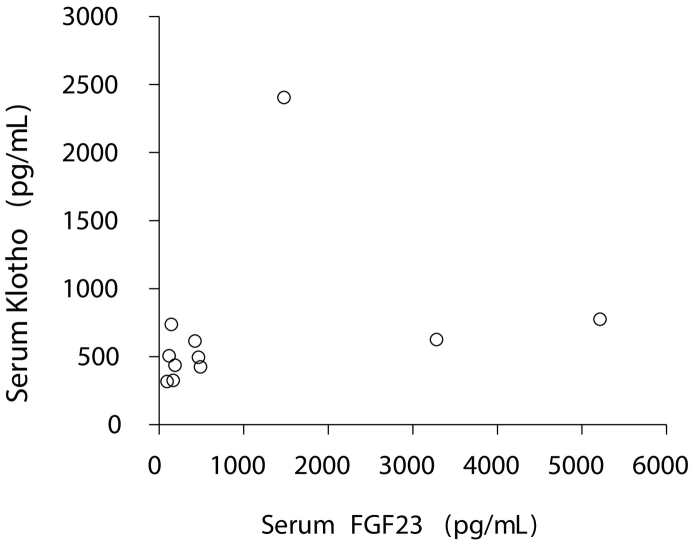
Relationships between serum FGF23 and Klotho concentrations in patients with TIO. There was no significant correlation between serum FGF23 and Klotho concentrations in patients with TIO (n = 11).

Serum Klotho concentrations in the 11 patients whose serum samples were available were between 317 and 2405 pg/mL (no reference range provided). Serum FGF23 concentrations in the corresponding patients were between 94 and 5211 pg/mL. There was no significant correlation between serum Klotho and serum FGF23 concentrations (r = 0.2332, *p* = 0.4902) (Fig. 4).

## 4 Discussion

In this study, we detected the ectopic expression of *Klotho* mRNA and Klotho protein in the FGF23-producing tumors that cause TIO. First, the result of RNA sequencing analysis showed the upregulation of *Klotho* mRNA in a tumor in a parotid gland compared to the adjacent normal tissue. Second, IHC analysis of the FFPE samples revealed positive Klotho staining in nine of 13 FGF23-producing tumors. A previous report by Yavropoulou et al. showed the expression of *Klotho* mRNA in an FGF23-producing tumor in the periphery of the fibula (Yavropoulou et al., 2015). In our study, we confirmed both the expression of *Klotho* mRNA and Klotho protein in the majority of tumors in various locations. When limited organs such as renal tubular cells express Klotho under physiological conditions, we hypothesize that the ectopic expression of Klotho in these mesenchymal tumors may be involved in the pathogenesis of TIO. Although the exact cause and the meaning of Klotho expression in PMTMCTs are not yet determined, we want to propose several hypotheses.

**Fig. 5:**
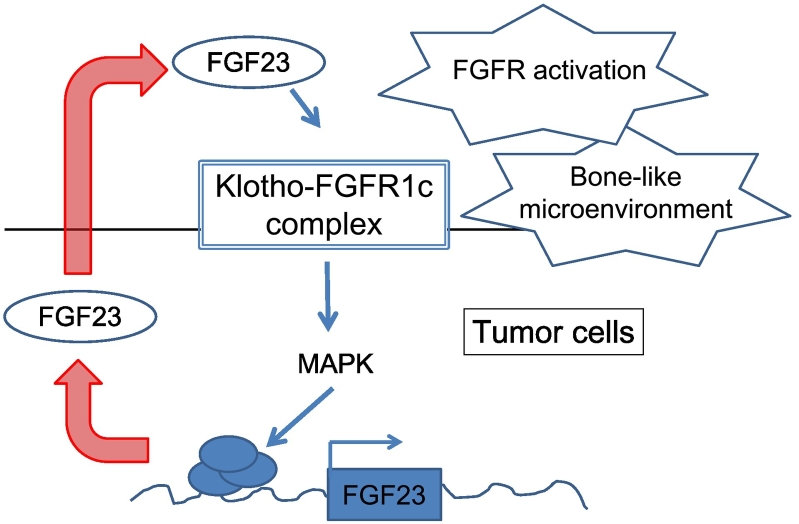
Autocrine/paracrine effects of FGF23 in FGF23-producing tumor cells. Membrane-bound Klotho enables the autocrine and paracrine effects of FGF23. FGF23 that is secreted from tumor cells binds to a receptor complex of Klotho and FGFR1c and activates the FGFR signaling pathway to enhance the production of FGF23 in tumor cells. The positive feedback loop in the production of FGF23 and a bone-like microenvironment in PMTMCTs may exacerbate the disease manifestations in patients with TIO. MAPK, Mitogen-activated Protein Kinase.

First, we conclude that the ectopic expression of Klotho in the tumor cells may lead to the activation of FGFRs. There is growing evidence that FGFRs not only serves as a receptor for FGF23 but also regulates FGF23 production. Wohrle et al. (2011) have shown that FGF9 induces *Fgf23* expression in the rat osteosarcoma cell line UMR-106 cells *in vitro*, which suggests that FGF23 is a target of FGFR signaling in bone. Osteoglophonic dysplasia (OGD; OMIM #166250) is caused by activating mutations in the *FGFR1* gene (White et al., 2005). It is reported that some patients with OGD develop FGF23-related hypophosphatemia in addition to various skeletal complications, such as craniosynostosis, dwarfism, and characteristic facial features (White et al., 2005). Conversely, conditional deletion of *Fgfr1* in osteocytes from *Hyp* mice, which is a murine model of XLH, has resulted in reduced circulating FGF23 (Xiao et al., 2014). In addition, pharmacological inhibition of FGFRs results in transient repression of *FGF23* mRNA in bone and a decrease in serum FGF23 concentrations in *Hyp* mice (Xiao et al., 2014), which is in turn followed by a compensatory increase in serum FGF23 levels after long-term therapy (Wohrle et al., 2013). Therefore, we hypothesize that the ectopic expression of Klotho in PMTMCTs helps in the activation of the FGFR signaling pathway and creates a local positive feedback loop for FGF23 production (Fig. 5). In other words, ectopically expressed Klotho enables the autocrine and paracrine effects of FGF23. The positive staining with phospho-Erk1/2 (Fig. 3) is in line with the activation of the FGFR signaling pathway in FGF23-producing tumor cells.

Second, we conclude that the expression of Klotho in PMTMCTs may reflect the osteoblastic differentiation of tumor cells. PMTMCTs are often present in the bone or the soft tissue adjacent to the bone, which makes it difficult to interpret the results of genetic analysis of such tumors. In our study, however, we used a tumor in the parotid gland, which is not mineralized in physiological conditions, to rule out the possibility of contamination by calcified tissues. Nonetheless, the result of RNA sequencing analysis showed the upregulation of genes that are related to osteoblasts and osteocytes (Table 2). Moreover, *matrix metalloproteinase 9* (*MMP9*) and *acid phosphatase 5*, *tartrate resistant* (*ACP5*), which are unique to osteoclasts and macrophages, were also upregulated. Since it has been shown that bone-forming cells such as osteoblasts and osteocytes express low amounts of Klotho (Raimann et al., 2013; Rhee et al., 2011; Komaba et al., 2017), the Klotho expression in tumor cells may reflect the differentiation of mesenchymal stem cells to osteoblastic lineage cells. Therefore, we hypothesize that a bone-like microenvironment is created in PMTMCTs. This bone-like microenvironment could augment FGF23 production in tumor cells.

Klotho, which is also referred to as αKlotho, is considered vital in the regulation of phosphate homeostasis, while βKlotho is required for FGF19 and FGF21 signaling (Kurosu and Kuro, 2009). There are different isoforms of Klotho protein: membrane-bound Klotho and soluble Klotho. Membrane-bound Klotho has a large extracellular domain that is subjected to ectodomain shedding and is released into the extracellular space as soluble Klotho (Matsumura et al., 1998). Several reports have suggested the involvement of soluble Klotho in the regulation of *FGF23* production. Brownstein et al. have reported a patient with FGF23-related hypophosphatemic rickets who harbored a translocation of a chromosome adjacent to the *Klotho* gene and elevated serum Klotho concentrations (Brownstein et al., 2008). Additionally, treatment with an adeno-associated virus that produced Klotho resulted in increased circulating serum Klotho and caused FGF23-related hypophosphatemia in mice (Smith et al., 2012). However, in our study, no correlation was found between serum Klotho and FGF23 concentrations. Therefore, we hypothesize that membrane-bound Klotho rather than soluble Klotho is involved in the mechanism of FGF23 production in PMTMCTs.

A previous study has shown that *Klotho* promoter lacks common regulatory elements such as TATA and CAAT boxes, and that DNA methylation of a CpG island in its promoter region appears to be responsible for the tissue-specific expression of the *Klotho* gene (Xu and Sun, 2015). We have conducted a methylation analysis using DNA extracted from tumors to see whether methylation status correlates with *Klotho* expression in FGF23-producing tumors. However, we found no correlation between promoter methylation status and the amount of *Klotho* mRNA expression (data not shown). Therefore, we conclude that DNA methylation does not principally regulate *Klotho* expression in FGF23-producing mesenchymal tumors.

The limitations of this study are as follows: First, as we used FFPE tissues instead of fresh frozen tissues for IHC analysis; the deterioration of samples over the years might have resulted in negative Klotho staining in some cases. Second, we could not determine whether the expression of Klotho and the presence of the *FN1-FGFR1* or *FN1-FGF1* fusion genes are mutually exclusive or not in FGF23-producing tumors. We did not detect these fusion genes for the four patients (cases #1, #3, #5, and #11) whose tumor RNAs were available. The presumed function of the protein products of the *FN1-FGFR1* and *FN1-FGF1* fusion genes is the activation of FGFR1 (Lee et al., 2016). Therefore, the presence of these fusion genes is not essential for tumors to produce FGF23 if there are alternative factors that activate FGFR1. In our case, the ectopic expression of Klotho and the overproduction of FGF1 (Table 2) may cause the activation of the FGFR1 signaling pathway. Finally, we could not determine the reason for MAPK activation in tumors without Klotho expression in our study (cases #6, #8, and #12 in Table 3). We have considered that false-negative Klotho staining because of sample deterioration, or the presence of *FN1-FGFR1* or *FN1-FGF1* fusion genes might explain the positive staining with p-Erk in Klotho negative samples. We will continue to study the mechanism of MAPK activation in PMTMCTs by using fresh tumor samples in the future.

In conclusion, we found the ectopic expression of Klotho in FGF23-producing tumors. Although the precise regulatory mechanism of Klotho expression in PMTMCTs is not clear, we hypothesize that Klotho helps to create a local positive feedback loop in the production of FGF23 through the activation of FGFR1 and exacerbates disease manifestations in patients with TIO. From a clinical perspective, the probable involvement of the FGFR signaling pathway in the pathogenesis of TIO justifies the application of FGFR inhibitors in patients with refractory TIO. Although the complete resection of tumors is always the optimal treatment for TIO, the results of our study suggest that patients without surgical indication suffering from unresectable, residual, or metastatic lesions may benefit from FGFR inhibitors.

## Conflict of interest

Dr. Kinoshita has received grants, KAKENHI 15K19528 and 17K16161, from Japan Society for the Promotion of Sciences (JSPS), during the conduct of the study; Dr. Takashi reports grants from JSPS, outside the submitted work; Dr. Ito reports grants from JSPS and research funding from Kyowa Hakko Kirin, outside the submitted work; Dr. Ikegawa has nothing to disclose; Dr. Mano has nothing to disclose; Dr. Ushiku has nothing to disclose; Dr. Fukayama has nothing to disclose; Dr. Nangaku reports advisory fees or research funding from Kyowa Hakko Kirin, Bayer Yakuhin, Torii Pharmaceutical, and Kissei Pharmaceutical, outside the submitted work; Dr. Fukumoto works in the laboratory supported by Chugai Pharmaceutical, Taisho Pharmaceutical, Ono Pharmaceutical, and Kyowa Hakko Kirin.

## Transparency document

Transparency document. Image 4
